# Are Bacterio- and Phytoplankton Community Compositions Related in Lakes Differing in Their Cyanobacteria Contribution and Physico-Chemical Properties?

**DOI:** 10.3390/genes12060855

**Published:** 2021-06-02

**Authors:** Mikołaj Kokociński, Dariusz Dziga, Adam Antosiak, Janne Soininen

**Affiliations:** 1 Department of Hydrobiology, Faculty of Biology, Adam Mickiewicz University, Uniwersytetu Poznańskiego 6, 61-614 Poznań, Poland; 2 Laboratory of Metabolomics, Faculty of Biochemistry, Biophysics and Biotechnology, Jagiellonian University, Gronostajowa 7, 30-387 Krakow, Poland; dariusz.dziga@uj.edu.pl (D.D.); adam.antosiak@doctoral.uj.edu.pl (A.A.); 3 Department of Geosciences and Geography, University of Helsinki, P.O. Box 64, FIN-00014 Helsinki, Finland; janne.soininen@helsinki.fi

**Keywords:** bacteria community, cyanobacteria, environmental variables, lakes, phytoplankton community, *Raphidiopsis raciborskii*

## Abstract

Bacterioplankton community composition has become the center of research attention in recent years. Bacteria associated with toxic cyanobacteria blooms have attracted considerable interest. However, little is known about the environmental factors driving the bacteria community, including the impact of invasive cyanobacteria. Therefore, our aim has been to determine the relationships between heterotrophic bacteria and phytoplankton community composition across 24 Polish lakes with different contributions of cyanobacteria including the invasive species *Raphidiopsis raciborskii.* This analysis revealed that cyanobacteria were present in 16 lakes, while *R. raciborskii* occurred in 14 lakes. Our results show that bacteria communities differed between lakes dominated by cyanobacteria and lakes with minor contributions of cyanobacteria but did not differ between lakes with *R. raciborskii* and other lakes. Physical factors, including water and Secchi depth, were the major drivers of bacteria and phytoplankton community composition. However, in lakes dominated by cyanobacteria, bacterial community composition was also influenced by biotic factors such as the amount of *R. raciborskii*, chlorophyll-a and total phytoplankton biomass. Thus, our study provides novel evidence on the influence of environmental factors and *R. raciborskii* on lake bacteria communities.

## 1. Introduction

The environmental factors that drive the phytoplankton and zooplankton community composition have been at the center of attention of scientists for many years. Studies have typically focused on local abiotic variables including temperature, light conditions, nutrient concentrations and their ratio, pH and conductivity, and they have also addressed biotic factors [1,2,3,4,5]. However, bacterioplankton assemblages as an integral part of biodiversity of the aquatic ecosystem have recently been attracting attention. In the past, bacteria were often considered as part of the microbial loop responsible for the demineralization of organic matter in the nutrient cycle and energy flow [3,6,7,8,9,10]. At present, bacteria associated with cyanobacteria, and colony forms in particular, have come into focus. Moreover, bacteria have been considered as environmentally friendly agents which mitigate cyanobacteria blooms and biodegrade cyanotoxins from water and sediments [11,12,13,14].

Much less is known about the entire lake bacterial community composition (BCC). However, the development of new molecular methods has made way for research on bacterial communities while the number of studies on bacterioplankton diversity in both local and regional scales has increased in recent years [15,16,17,18]. Bacterioplankton community compositions have been related to various environmental drivers including interactions with phytoplankton [15,19], nutrient availability [20,21] and physical disturbances [22]. Bacteria and cyanobacteria are closely related to many important chemical processes occurring in aquatic ecosystems, including ammonification, nitrification and denitrification [23]. Moreover, our recent study has shown some specific relations between microcystin producers and specific microcystin degraders belonging to several bacteria genera [24]. Nonetheless, little is known about environmental factors driving bacterioplankton, especially about the relationship between phytoplankton and bacterioplankton composition. This is especially true in lakes dominated by cyanobacteria and across larger sets of lakes that would provide a more general view on the bacteria–algae relationship [25]. In addition, very limited research have reported how alien cyanobacteria may impact the bacterioplankton community composition, including the highly expansive cyanobacterium *Raphidiopsis raciborskii* [26]. It originates from tropical and subtropical regions, however, its successful expansion toward temperate zones has been observed over the past decades. The increasing number of reports of *R. raciborskii* in temperate zones and its increasing contribution in the phytoplankton biomass suggests the invasive character of this cyanobacterium. While several theories explaining its invasiveness have been proposed, including climate changes, phenotypic plasticity or the occurrence of highly adaptive strains within a population, a comprehensive answer is still not known. Our recent paper provided an initial insight into the genetic and physiological aspects involved in the strain-specific adaptation to temperate climates [27]. Moreover, some strains of *R. raciborskii* are proven to be capable of cyanotoxins production but the impact of this cyanobacterium on invaded aquatic ecosystems is poorly understood [28].

Therefore, based on the further re-analysis and re-interpretation of data from our earlier study [24], our aims have been: (i) to determine bacteria and phytoplankton composition across the studied lakes; (ii) to compare bacteria community composition between: (a) lakes dominated by cyanobacteria (hereafter: CyanoDominantLakes) and those with minor contributions of cyanobacteria (hereafter: CyanoMinorLakes) and (b) lakes with a presence or absence of *R. raciborskii*; (iii) to identify which environmental factors drive the microbial community composition; and finally, (iv) to verify if bacterioplankton and phytoplankton communities show any congruence in their community compositions in the studied lakes. The lakes included in the research are of post-glacial origin, from shallow to deep, of various trophic status, and have a catchment area that is predominantly agricultural.

## 2. Materials and Methods

### 2.1. Collection of Water Samples

This study has been based on the samples collected previously [24], in 24 lakes randomly selected in the western part of the Wielkopolska and Kujawsko-Pomorskie regions in Poland. From each lake, one sample was collected from its central part between July and September 2016 (8 samples) and 2017 (16 samples). The lakes varied in morphometry, mixing regime and trophic status representing meso- to highly-eutrophic ecosystems. Geographical position, limnological characteristics and physico-chemicals properties of the lakes have been provided in Table S1. Phytoplankton samples were collected from the surface layer of a water column in polymictic lakes or epilimnion in stratified lakes using a 5 L bathometer (UWITEC, Austria). After mixing, 1 L subsamples for chemical and phytoplankton analysis were collected and transported to the laboratory. The phytoplankton samples were immediately preserved with acidified Lugol’s solution with a final concentration of 1% (APHA 1998) and stored in cool and dark conditions until they were analyzed.

### 2.2. Phytoplankton Analysis and Physicochemical Parameters

Prior to their analysis, the phytoplankton samples were left in 1 L glass cylinders for 48 h, following which, the overlying water was gently decanted off and the sedimented material in the lower layer (volume 20–40 mL) was used for phytoplankton analysis. Phytoplankton was identified and counted in 100–150 fields of the Fuchs–Rosenthal chamber using an Olympus BX 60 light microscope under 400× magnification. At least 400 specimens were counted to reduce the error to less than 10%. A single cell, a coenobium or a filament represented one specimen in the analysis. Phytoplankton biomass was estimated from the biovolume, calculated for each species based on the volume formulae of geometric solids most closely resembling a given species, according to Hillebrand et al. [29], verified with the recent adjustments [30] and expressed as wet weight [31].

The concentrations of nitrate (DN) and ortophosphate (DP) were determined by a spectrophotometric method. For a chlorophyll-a analysis, 200–500 mL of water was filtered through a GF/C Whatman filter. The concentration was determined spectrophotometrically after extraction with 90% acetone and calculated using Lorenzen’s formula [31]. In the course of sampling, water temperature, pH and conductivity were determined in the field using a multiparameter probe and the Secchi depth (SD) was measured.

### 2.3. Bacterioplankton Community Composition

The metagenomic procedure used to determine the bacterial community composition was described earlier by Dziga et al., including supplementary files [24]. Briefly, we constructed 16S libraries from DNA extracted from the material deposited on 8 μm 0.45 μm sterile cellulose nitrate filters during filtration of 500 mL of water sample. DNA was isolated using a FastDNA spin kit for soil (MP Biomedicals) and sequencing libraries were prepared by amplifying the V3 region of the 16S rRNA gene (~150–194 bp; [32]). For primers, protocols and the conditions, see supplement S1 from the previous publication [24,33]. Amplicons were sequenced using an Ion 314 chip (samples collected in 2016) or an Ion 318 chip (samples collected in 2017) using a standard workflow.

Libraries were barcode-sorted and quality-filtered using BBDuk (read fragments with scores below 20 were trimmed and sequences shorter than 100 were discarded). Chimeras were removed with the ‘identify chimeric seqs’ from the QIIME 1.9.1 suite. The clean data were processed using QIIME de novo OTU (Operational Taxonomic Unit) picking workflow set on default run (Caporaso et al., 2010; 97% Uclust similarity cut-off, Greengenes database v13.8). The pipeline assigned taxonomy and constructed an OTU table. The sequence read files have been deposited in the BioSample database with accession numbers PRJNA730760 (https://www.ncbi.nlm.nih.gov/sra/PRJNA730760, accesed 6 May 2021).

### 2.4. Statistical Analysis

An analysis of similarities (ANOSIM) was used to determine if bacteria communities differ between CyanoDominantLakes (lakes where the contribution of cyanobacteria is over 50% of the total phytoplankton biomass) and other lakes, and between lakes containing *R. raciborskii* and lakes where this species does not occur. We then used the redundancy analysis (RDA) to explore the main patterns of bacterio- and phytoplankton community composition and to relate these to the measured environmental variables in all the lakes. Finally, we also used the Mantel test with the Pearson correlation coefficient to examine whether the bacterio- and phytoplankton communities showed significant concordance in their community composition. Significant concordance would indicate the following: if two lakes harbor, for instance, similar bacterioplankton composition, they would also harbor similar phytoplankton composition. The Mantel test was conducted separately for all the studied lakes, cyanobacteria lakes and lakes containing *R. raciborskii.* Prior to the RDA analysis, biotic data were Hellinger-transformed and environmental data log-transformed, except water pH.

To determine the relation of nitrifying and denitrifying bacteria to cyanobacterial biomass, the OTUs of phylum cyanobacteria were used as an internal standard. Thus, the relative abundance of these groups was converted into quantitative data. Due to non-normal distribution, a Mann–Whitney test was conducted to compare the content of bacteria involved in nitrogen metabolism based on the abundance of families that includes species capable of nitrification (*Nitrospiraceae*, *Bradyrhizobiaceae* and *Nitrosomonadaceae*) and denitrification (*Actinomycetaceae*, *Corynebacteriaceae*, *Mycobacteriaceae*, *Propionibacteriaceae*, *Flavobacteriaceae*, *Bacillaceae*, *Staphylococcaceae*, *Lactobacillaceae*, *Rhodobacteraceae*, *Sphingomonadaceae*, *Burkholderiaceae*, *Neisseriaceae*, *Rhodocyclaceae*, *Bacteriovoracaceae*, *Bdellovibrionaceae*, *Geobacteraceae*, *Syntrophobacteraceae*, *Campylobacteraceae*, *Shewanellaceae*, *Pseudomonadaceae*, *Leptospiraceae*). The selection of families from the denitrification groups was made in accordance with [23]. Additionally, the correlation between the cyanobacteria and the nitrifying bacteria was verified using the Pearson correlation test. R software (R Core Team 2017) and Statistica 13 (TIBCO Software Inc., Palo Alto, CA, USA, 2017) were used for data analyses.

## 3. Results

### 3.1. Physico-Chemical Characteristics

Physico-chemical features varied significantly across the investigated lakes. Altogether, 19 out of the 24 studied lakes showed eutrophic status with visibility below 1 m, alkaline character with pH ranging from 7.7 to 9.0 and high conductivity from 505 to 870 μS cm^−1^. Chlorophyll-a ranged broadly from 3.6 to 184.7 µg L^−1^ across the lakes with concentrations higher than 50 µg L^−1^ in 19 lakes (Table S1).

### 3.2. Phytoplankton Composition

As expected, cyanobacteria were the dominant phytoplankton group in 16 lakes with a biomass contribution in the total phytoplankton biomass of over 50% (CyanoDominantLakes). In the remaining eight lakes, the contribution of cyanobacteria biomass did not exceed 50% of the total phytoplankton biomass (CyanoMinorLakes) (Table 1 and Table S2). The most common cyanobacteria were *Planktothrix agardhii*, *Aphanizomenon gracile*, *Limnothrix* sp., *Jaaginema subtilissimum*, *Planktolyngbya limnetica* and *Pseudanabaena limnetica*. *P. agardhii* was the most abundant cyanobacterium in the majority of the studied lakes. Moreover, in 10 lakes it accounted for 7 to 87% of the total phytoplankton biomass. The second most abundant cyanobacterium was *Limnothrix* sp. from Synechococcales group, with a percentage in total phytoplankton biomass ranging from 0 to 83%. Among heterocystous cyanobacteria, the most common was *Aphanizomenon gracile*, detected in 20 lakes with a contribution in total phytoplankton biomass up to 79%. The invasive cyanobacterium *R. raciborskii* occurred in 14 lakes (hereafter: R.raciborskiiLakes) that includes freshwaters belonging mainly to CyanoDominantLakes with a biomass ranging from 0.02 to 10 mg L^−1^ and contributions of up to 30% in the total phytoplankton biomass (Table 1 and Table S2). The most abundant phytoplankton groups in the CyanoMinorLakes were Chlorophyceae (*Tetraëdron minimum*, *Tetrastrum glabrum*, *Phacotus lenticularis*), Bacillariophyceae (*Aulacoseira granulata*, *Ulnaria acus*, *Ulnaria ulna*), Dinophyceae (*Peridiniopsis cunningtonii*, *Peridinium cinctum*) and Euglenophyceae (*Phacus* sp.). However, their contribution varied among the lakes (Table S2).

### 3.3. Bacterioplankton Community Composition

**Figure 1:**
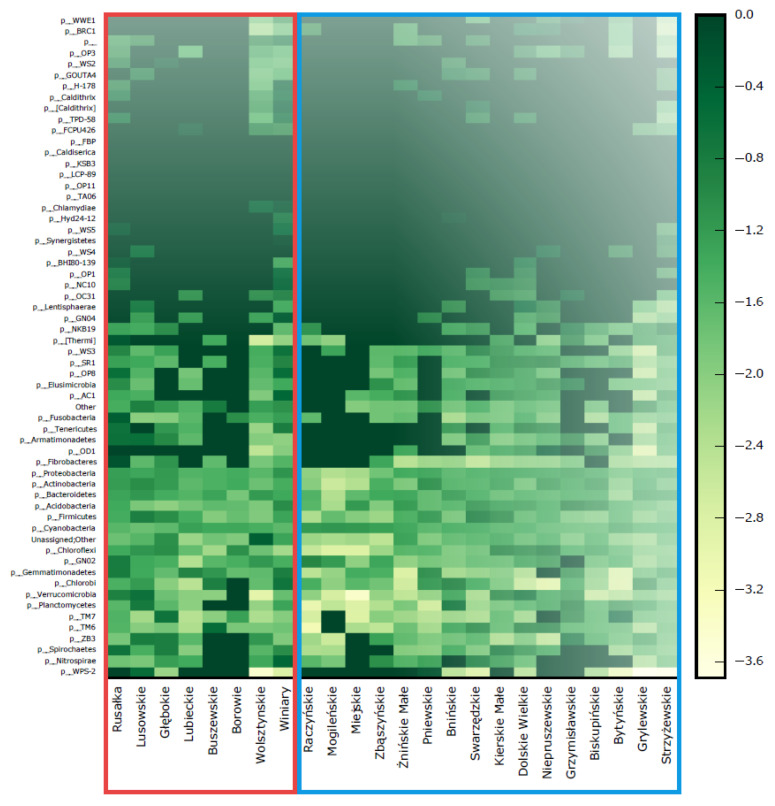
The heat map of bacterioplankton content (at the phylum level) in CyanoMinorLakes (red) and CyanoDominantLakes (blue).

A set of 987 OTUs was defined at 97% sequence similarity. A total of 51 phylum-, 124 class-, 196 order-, 245 family- and 374 genus-level bacterial taxonomic units (part of these taxa have not been yet officially accepted) were identified in the 24 investigated lakes. In total, *Proteobacteria*, *Actinobacteria* and *Bacteroidetes* contributed up to 90% of the relative abundance of the bacteria community (Figure 1). The bacteria from the other detected phyla including *Firmicutes*, *Chloroflexi*, *Chlorobi* and *Acidobacteria* were less abundant. Within bacteria families, *Flavobacteriaceae*, *Cryomorphaceae*, *Saprospiraceae*, *Cyclobacteriaceae (Bacteroides)*, *Pelagibacteraceae*, *Acetobacteraceae*, *Comamonadaceae*, *Methylococcaceae*, *Oxalobacteraceae*, *Xanthobacteraceae* (*Proteobacteria*) and *Microbacteriaceae* (*Actinobacteria*) had the highest average relative abundance in the studied lakes.

The most abundant bacterial genera were: Methylocaldum, Rhodobacter, Novosphingobium, Crenothrix, Phenylobacterium (Proteobacteria), Flavobacterium (Fluviicola), Sediminibacterium (Bacteroidetes), Mycobacterium (Actinobacteria). The abundance of chloroplast sequences varied from 1 to 10% of the cyanobacterial sequences.

The average number of OTUs in the CyanoMinorLakes (366) was slightly higher than in the CyanoDominantLakes (312). In contrast, the average number of OTUs in the R.raciborskiiLakes (313) was lower than in lakes without this cyanobacterium (354).

### 3.4. Community Patterns and Drivers

According to the ANOSIM analysis, bacterioplankton communities differed between the CyanoDominatLakes and the CyanoMinorLakes (R = 0.175, *p* = 0.031), but did not differ significantly between the R.raciborskiiLakes and the other lakes (R = 0.080, *p* = 0.121).

**Figure 2:**
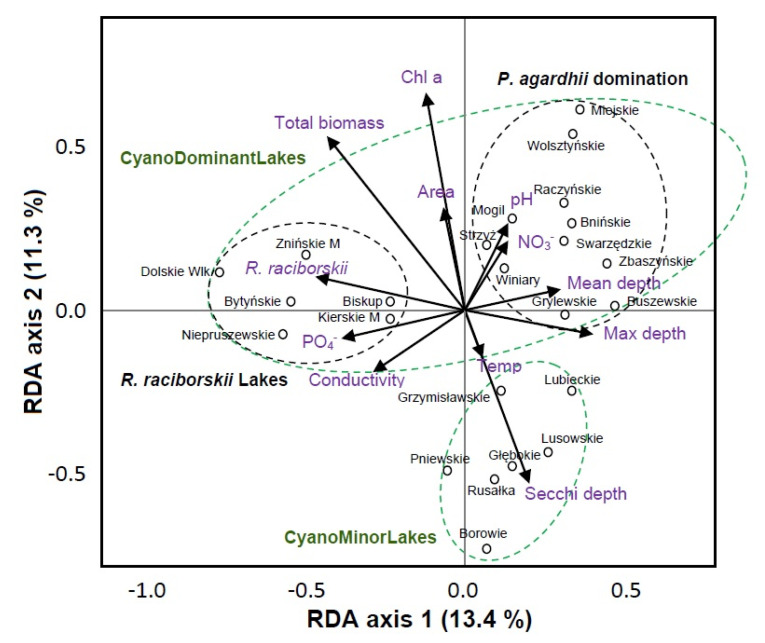
Redundancy analysis plot for bacterioplankton in the studied lakes divided into two main groups. The variation explained by each axis has been plotted on the *x*-axis (RDA 1—eigenvalue 0.030) and *y*-axis (RDA2—eigenvalue 0.026).

The main contributors for the RDA axis 1 for bacterioplankton were water depth and the amount of *R. raciborskii* (Figure 2). The second axis (RDA 2) was comprised mostly of chlorophyll-a, total phytoplankton biomass and the Secchi depth. In RDA, we could explain 59.6% of the total variation in the bacteria community composition in the lakes. The RDA analysis grouped the lakes into three categories: lakes with *R. raciborskii* (R.raciborskiiLakes), lakes dominated by *P. agardhii* and lakes with low phytoplankton biomass (both total and cyanobacterial) (Figure 2). The first two groups include most of the lakes from the CyanoDominantLakes category while the third group includes the lakes classified as CyanoMinorLakes.

**Figure 3:**
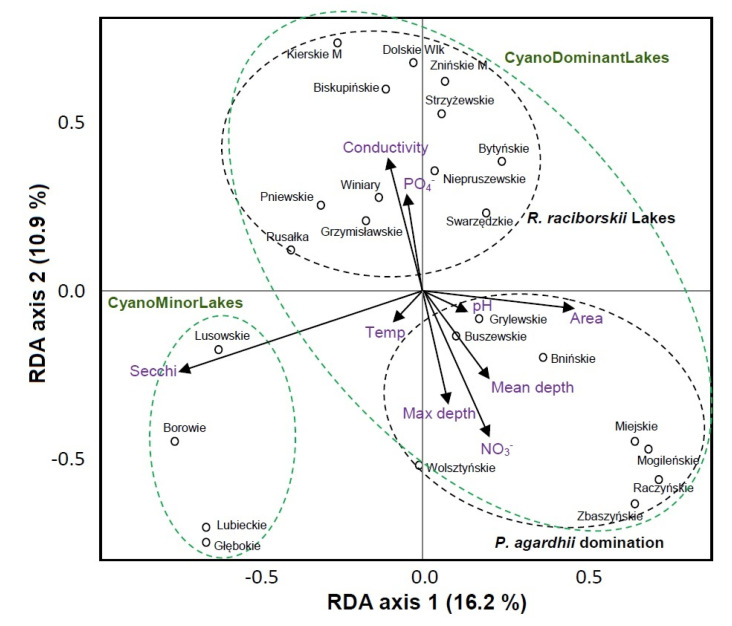
Redundancy analysis plot for phytoplankton in the studied lakes divided into two main groups. The variation explained by each axis has been plotted on the *x*-axis (RDA 1—eigenvalue 0.103) and *y*-axis (RDA2—eigenvalue 0.070).

The RDA for phytoplankton indicated that the first main gradient (RDA 1) was best related to Secchi depth and lake area (Figure 3). The second main gradient RDA 2 was best related to conductivity, water depth and nitrate (NO_3_^−^). In RDA, we could explain 49.2% of the total variation in the phytoplankton community composition in the lakes. Similarly to bacterioplankton, the RDA distinguished three categories of lakes comprising roughly the same number as in the bacterioplankton grouping. Likewise in bacterioplankton, the first two groups include most of the lakes from the CyanoDominantLakes category while the third group includes the lakes classified as CyanoMinorLakes (Figure 3).

According to the Mantel test, bacterio- and phytoplankton communities were not related significantly in the CyanoDominantLakes (R = 0.020, *p* = 0.345), in all the studied lakes (R = 0.045, *p* = 0.291) or in the lakes with *R. raciborskii* (R = 0.046, *p* = 0.535).

**Figure 4:**
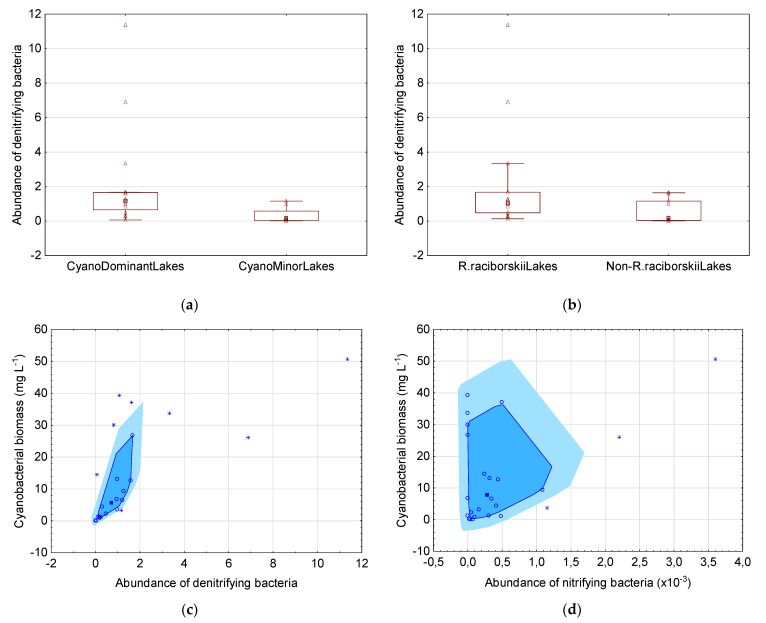
The abundance of families harboring denitrifying bacteria in different groups of lakes (**a**,**b**) and the correlation between total biomass of cyanobacteria and denitrifying (**c**) and nitrifying bacteria (**d**). In the box plot, the median has been shown as the square, the range 25–75% as the box, the non-outlier range as whiskers, and the raw data as triangles (**a**,**b**). On the bagplot the median has been presented as the square, the raw data as circles and outliers as asterisks (**c**,**d**).

The content of denitrifying bacteria was markedly higher (6.3-fold, *p* < 0.01) in the CyanoDominantLakes as compared to the CyanoMinorLakes (Figure 4a). The higher abundance of nitrifying bacteria (2.3-fold) was also observed in CyanoDominantLakes but due to high variability the statistically important differences were not documented (Figure 4b). The R.raciborskiiLakes contained similar amount of both nitrifying and denitrifying bacteria in comparison with the lakes where *R. raciborskii* was absent or below 1% of the total phytoplankton biomass (data not presented). A positive correlation was found between the cyanobacterial biomass and the content of both nitrifying (r = 0.46, *p* < 0.05) and denitrifying (r = 0.69, *p* < 0.05) bacteria (Figure 4c,d).

## 4. Discussion

The aim of our study has been to determine the bacteria and phytoplankton community composition across lakes varying in their physical and chemical conditions and to identify the impact of both abiotic and biotic factors on the diversity of bacterial and phytoplankton composition. We initially assumed that the bacteria and phytoplankton community composition might be closely related across the lakes and that there are correlations between invasive cyanobacteria and heterotrophic bacteria assemblages. Our data generally showed that bacteria communities differed between lakes dominated by cyanobacteria (CyanoDominantLakes) and lakes with minor contribution of cyanobacteria (CyanoMinorLakes), but bacterio- and phytoplankton communities were not related significantly in any type of lakes. Moreover, the invasive cyanobacterium *R. raciborskii* did not affect bacterial communities while physical factors were the major drivers of bacteria and phytoplankton community composition.

### 4.1. Environmental Factors Driving the Bacteria and Phytoplankton Community Composition

The RDA indicated (Figure 2) that the bacteria were influenced to some degree by biotic factors such as the presence and amount of phytoplankton in terms of the chlorophyll-a concentration and the total phytoplankton and *R. raciborskii* biomass in the CyanoDominantLakes. Despite this, abiotic factors such as water depth and Secchi depth had some impact on the structure of bacteria community in CyanoMinorLakes. The impact of high chlorophyll-a and total biomass complies with the results presented by Pineda-Mendoza et al. [18], who reported significant negative influence of high phytoplankton biomass dominated by *Microcystis* spp. on the richness and diversity values of heterotrophic bacteria. On the other hand, at a low nutrients concentration during the bloom, cyanobacterial biomass could serve as a plausible nutrient source for heterotrophic bacteria [17].

In the case of phytoplankton, the RDA indicated (Figure 3) that physical factors including the Secchi depth, lake area and water depth regulated mainly the phytoplankton communities. However, the Secchi depth was negatively related to phytoplankton communities in the CyanoMinorLakes while water depth and lake area were negatively related to *P. agardhii* lakes from the CyanoDominantLakes category. The latter finding complies with the environmental demands of *P. agardhii* that dominates shallow and rather small, turbid lakes. The phytoplankton community in the R.raciborskiiLakes, the third group distinguished in the RDA for phytoplankton, was related to conductivity (positively) and nitrate concentration (negatively) (Figure 3). This complies with the findings by Kokociński et al. [34] who reported conductivity among the factors that had the biggest impact on *R. raciborskii* occurrence.

### 4.2. The Bacteria and Phytoplankton Community Composition across Different Types of Lakes and the Relations within Microbial Communities

The present research has indicated that the phytoplankton community composition in the CyanoDominantLakes reflects the typical structure characteristic for lakes with high trophic status dominated by various cyanobacteria species. Among them, *P. agardhii* was the most abundant and was found in the majority of these lakes (Table 1 and Table S2). These results are similar to other observations that this species commonly occurs in shallow, polymictic lakes with high nutrient concentrations [35,36]. Even more common, however, with lower contribution to total phytoplankton biomass was *R. raciborskii*, which confirms its progressive expansion and growing potential in plankton communities (Table 1 and Table S2).

The documented domination of Proteobacteria and Actinobacteria is in line with findings from other studies carried out in Poland [37] as well as in urban lakes in Mexico—a geographically remote region [18]. Additionally, the occurrence of *Proteobacteria* and *Actinobacteria* as dominant groups in lakes has been frequently associated with cyanobacterial blooms [38].

The dominant genera including *Flavobacterium*, *Rhodobacter* and *Hydrogenophaga* were also similar to the latter studies, which indicate a worldwide occurrence and contribution of these taxa in bacterioplankton communities. Moreover, some previous studies showed the dominance of *Protebocateria* and *Actinobacteria* in lakes dominated by cyanobacteria from the *Microcystis* genera [39,40] while the ecosystems investigated in the present study were dominated by *P. agardhii*. This may indicate that these bacteria groups are commonly associated with cyanobacterial blooms caused by different species.

Recently several papers employing high-throughput sequencing (HTS) have been published and report changes in the diversity of both bacterial and cyanobacterial communities, focusing on one or several lakes in the whole season. Regarding diversity, our ANOSIM analysis showed that bacterioplankton communities differed between the CyanoDominantLakes and the CyanoMinorLakes, with a slightly higher average number of OTUs and bacteria genera in the latter group. Furthermore, several reports also appear regarding the impact of trophic status on bacterial community composition. Such relations between BCC and trophic status were reported by Pineda-Mendoza et al. [18]. They documented that under highly eutrophic conditions a reduced number of species can exist, making communities less diverse, which results in the dominance of only few major taxa. In our previous study [24], *P. agardhii*, a dominant species in some lakes, was found to negatively impact the abundance of bacteria. This suggests that its domination reduces bacterial diversity. In addition, Koo et al. [41] observed a negative impact of high nutrient concentrations and poor water quality on microbial diversity. In contrast, Song et al. [42] reported that summer cyanobacterial blooms are associated with increased abundance and diversity of heterotrophic bacteria. Thus, in the freshwaters dominated by cyanobacteria, both positive and negative impacts of dominant cyanobacteria on the bacteria diversity occur, resulting in the overall modification of the BCC.

To our knowledge, this report is the first to provide a comparison of bacterial consortia between lakes with and without *R. raciborskii*. An intriguing finding is that in the lakes inhabited by *R. raciborskii*, which accounted for up to 30% of the phytoplankton community, the BCC was similar to those lakes where *R. raciborskii* did not occur. Overall, it suggests that a high abundance of *R. raciborskii* does not influence dominant OUTs. This is supported by an earlier study conducted by Bouvy et al. [43], where total bacterial abundance was not significantly correlated with the variations of *R. raciborskii* biomass in Ingazeira reservoir in Brazil. An interesting report from Woodhouse et al. [44] showed that despite a clear seasonal modification of both cyanobacterial and bacterial composition, cyanobacteria are more likely correlated with stable, dominant heterotrophic taxa that contributed especially to the overall microbial community during the more stable summer period. This indicated a stable association of most dominant cyanobacteria and bacteria. As was concluded by Burke et al. [45], dominant bacteria may possess a particular metabolic potential allowing for a more efficient proliferation at the beginning of the vegetative season, which restricts the ability of other genera to establish a higher abundance. Therefore, the impact of cyanobacteria (such as *R. raciborskii*) that colonize the freshwater ecosystems in late season on the BCC may be limited. On the other hand, the lack of differences in the structure of bacterioplankton communities between lakes with and without *R. raciborskii* may result from the low contribution of this cyanobacterium in the total phytoplankton biomass in many studied lakes. However, as shown by RDA analysis, the biomass of *R. raciborskii* was one of the factors influencing bacterioplankton in lakes with a higher share of this cyanobacterium.

According to Mantel tests, bacteria and phytoplankton communities were not related significantly, either in the whole set of the investigated lakes or in the CyanoDominantLakes or the R.raciborskiiLakes. Usually, a high temporal variability in the cyanobacterial assemblage at various bloom stages is correlated with a changed bacterial composition [26,46]. In natural habitats, populations of different cyanobacteria co-exist, creating microenvironments for other microorganisms. Therefore, several diverse bacterial phyla form an integral part of the algal blooms’ diversity [47,48]. Both positive and negative interactions between the existing species occurs [49]. However, cyanobacterial abundance and diversity of the microbial community are not always related [50], as in our study.

Moreover, the relationships between the occurrence of certain cyanobacteria, for example, from the *Microcystis* genera and bacteria community structure, were frequently reported [39,46,51] and therefore the impact of the invasive *R. raciborskii* on co-occurring bacteria should not be neglected. There are, however, very limited data (based only on sampling from individual freshwater ecosystems) on the association of bacterial community with *R. raciborskii*. Guedes at al. [50] observed the specific interactions of the dominant cyanobacterial genera with some heterotrophic bacteria in a tropical reservoir in Brazil. The second stage of the bloom, dominated by *R. raciborskii* and *Synechococcus* spp., was associated with a shift in the bacteria composition and some Planctomycetes OTUs were positively correlated with this species.

### 4.3. Congruence in the Composition of Microbial Community in the Context of Nitrification and Denitrification

The association of bacteria involved in nitrogen metabolism with freshwater cyanobacteria is another important aspect of the interaction within microbial community. Interesting relations between cyanobacteria and bacterial genera (*Pseudomonas* and *Bacillus*) harbouring denitrifying species in West Lake were found by Song et al. [42]. An increased abundance of these bacteria was correlated with the level *nirK* and *nirS* genes involved in nitrite reduction. It has been suggested that the denitrification process in surface water (when oxygenic conditions occur) may provide molecular nitrogen for diazotrophic cyanobacteria, thus supporting their growth and competition with other genera. In contrast, the results of Qian et al. [52] indicated positive correlation of denitrifying bacteria with non-diazotrophic *Microcystis* sp. In Lake Taihu. The authors concluded that although such bacteria releasing N_2_ may reduce the nitrate availability for, e.g., *Microcystis*, in freshwaters rich in N sources, such competition for this nutrient may have limited impact on the cyanobacterial growth and bloom formation. Our results are in agreement with both these studies cited above. A significantly higher level of denitrifying bacteria in CyanoDominantLakes and their positive correlation with total cyanobacterial biomass (Figure 4) suggest that this type of nitrogen metabolism (denitrification) may play a crucial role in the distribution of diazotrophic and non-diazotrophic cyanobacteria genera present in the investigated ecosystems. On the other hand, the positive correlation of families that includes nitrifying bacteria genera with cyanobacterial biomass (Figure 4) are in contrast with the research of Cui et al. [53]. The role of nitrifying species in the succession within the microbial community has been emphasised by these authors who explained the reduced bloom formation by the redirection of inorganic nitrogen flow. As was concluded, organic nitrogen released from the cyanobacteria detritus may be further degraded by nitrifying bacteria, which produce inorganic nitrogen resulting in increased total dissolved nitrogen. This source can be again incorporated into organic matter by different players, not only by cyanobacteria. Our results deny this hypothesis and suggest that the production of nitrite and nitrate may bring benefits also to cyanobacteria.

## 5. Conclusions

The mechanisms influencing the formation of cyanobacterial blooms are not only attributed to the competition for nutrients, but also to the interaction with other components of the microbial community. The presented results are based on a broad-scale study conducted to determine the relationships between heterotrophic bacteria and phytoplankton community composition across a variety of lakes with different contribution of cyanobacteria and the invasive cyanobacterium *Raphidiopsis raciborskii.* We have shown that bacteria and phytoplankton communities differ between lakes dominated by cyanobacteria and lakes with a minor contribution of cyanobacteria. The impact of this invasive cyanobacterium on bacteria community has been examined for the first time. The abundance of phytoplankton in terms of *R. raciborskii* biomass, chlorophyll-a concentration and total phytoplankton biomass was found to have impact on bacteria communities in lakes dominated by cyanobacteria. In turn, abiotic factors including water depth and Secchi depth were the major drivers of bacteria and phytoplankton community composition. Furthermore, we demonstrated a higher abundance of the bacteria families that harbor both the denitrifying and nitrifying genus in lakes dominated by cyanobacteria.

Considering both abiotic and biotic interactions may lead to a more comprehensive understanding of cyanobacterial dominance. Better cognition of microbial interactions should provide a novel insight into the development of control strategies which allow more efficient predictions and thus suppression of cyanobacterial growth in an environmentally-friendly manner.

## Figures and Tables

**Table 1:** The contribution of cyanobacteria and two main species (*P. agardhii* and *R. raciborskii*) expressed as a percentage of total phytoplankton biomass.

Lake	Total Phytoplankton Biomass [mg L^−1^]	Cyanobacteria Biomass [mg L^−1^]	Cyanobacteria Biomass [%]	*Planktothrix agardhii* Biomass [%]	*Raphidiopsis raciborskii* Biomass [%]
**CyanoDominant** **Lakes**					
Kierskie Małe	11.4	6.5	56.9	0	8.3
Zbąszyńskie	16.9	13.0	76.8	73.9	0
Niepruszewskie	33.7	25.9	76.8	1.3	1.5
Bnińskie	11.4	6.7	58.8	46.6	2.2
Raczyńskie	41.8	39.2	93.7	87.5	0.07
Grzymisławskie	2.3	1.1	51.1	0	5.0
Dolskie Wielkie	36.0	33.7	93.3	0	30.1
Żnińskie Małe	30.4	26.7	87.7	0.4	0.6
Mogileńskie	15.2	14.3	94.3	82.2	0
Miejskie	33.6	29.9	88.9	71.9	4.3
Pniewskie	7.1	4.3	61.1	1.7	5.0
Bytyńskie	39.5	37.0	93.5	7.1	0
Swarzędzkie	14.5	9.3	64.4	7.8	0.2
Strzyżewskie	16.0	12.9	78.5	3.0	0
Biskupińskie	55.3	50.5	91.4	1.2	0.05
Grylewskie	2.9	2.2	75.4	11.7	0.6
**CyanoMinor** **Lakes**					
Buszewskie	3.2	1.3	40.6	8.6	0.8
Lubieckie	1.3	0.2	16.7	0	0
Borówie	3.0	1.2	40.7	0	0
Głębokie	2.2	0.07	3.4	0	0
Winiary	8.5	3.7	41.9	0	0.3
Rusałka	5.5	0.8	15.2	0	0
Lusowskie	0.17	0.04	23.6	0	0
Wolsztyńskie	11.9	3.1	26.7	15.2	0

Lakes containing *R. raciborskii* (R.raciborskiiLakes) are highlighted in red color.

## Data Availability

Data supporting reported results can be found at Faculty of Biology, Adam Mickiewicz University.

## Supplementary Materials

The following are available online at https://www.mdpi.com/article/10.3390/genes12060855/s1. Table S1: Physico-chemical characteristic of investigated lakes; Table S2: Phytoplankton content in the investigated lakes.
