# Supplementary material for: Are Bacterio- and Phytoplankton Community Compositions Related in Lakes Differing in Their Cyanobacteria Contribution and Physico-Chemical Properties?
Source: Genes (Basel). 2021 Jun 2;12(6):855. doi: 10.3390/genes12060855 (PMC8227929; doi:10.3390/genes12060855)
Supplement: Supplementary file 1 [file genes-12-00855-s001.zip › Table S1.pdf]

Lake	Surface [ha]	Mean depth [m]	Max. depth [m]	pH	Secchi depth [m]	Cond. [μS cm^{-1}]	Temp. [°C]	Chlorofil a [μg L^{-1}]	N-NO3 [mg L^{-1}]	P-PO4 [mg L^{-1}]
**CyanoDominantLakes**										
Kierskie Małe	26.0	1.4	2.3	8.74	0.6	852	16.8	101.04	0.06	0.13
Zbąszyńskie	742.5	3.5	9.6	8.56	0.4	755	16.2	101.31	0.19	0.001
Niepruszewskie	227.5	3.1	5.2	8.65	0.6	722	17.5	35.92	1.1	0.09
Bnińskie	221.5	4.0	8.5	8.33	0.8	663	16.8	105.49	0.05	0.08
Raczyńskie	93.5	2.7	5.8	8.9	0.2	505	16.8	107.32	0.08	0.2
Grzymisławskie	180.1	3.0	11.2	8.38	0.9	870	17.8	80.72	0.04	0.07
Dolskie Wielkie	166.6	1.2	3.0	8.98	0.4	642	18.5	77.92	0.07	0.12
Żnińskie Małe	122.5	2.3	5.3	8.9	0.45	706	22.7	150.16	0.04	0.12
Mogileńskie	72.5	3.2	6.8	9.02	0.4	585	24.5	93.23	0.96	0.001
Miejskie	44.0	1.5	6.4	8.97	0.4	537	25.6	108.68	0.24	0.001
Pniewskie	55.0	1.5	3.3	8.79	0.55	508	24.7	30.47	0.001	0.001
Bytyńskie	308.8	3.5	7.5	7.78	0.5	786	24.3	73.16	0.04	0.001
Swarzędzkie	94.0	2.3	6.5	8.8	0.5	639	19.8	184.75	0.05	0.001
Strzyżewskie	48.9	5.8	13.3	8.51	0.7	632	19.1	69.49	0.06	0.001
Biskupińskie	107.4	5.5	13.5	8.71	0.7	775	20.3	72.29	0.04	0.001
Grylewskie	98.0	3.6	6.5	8.42	0.7	832	22.7	53.11	2.3	0.001
**CyanoMinorLakes**										
Buszewskie	79.8	4.8	14.0	9.04	0.8	601	24.5	57.69	0.04	0.001
Lubieckie	47.5	4.9	8.6	8.74	1.7	559	25.2	7.33	0.2	0.001
Borówie	5.0	2.4	5.0	8.49	1.1	505	23	13.11	0.03	0.001
Głębokie	25.2	2.1	6.0	8.43	1.7	803	20.9	15.77	1.32	0.02
Winiary	19.0	3.5	6.0	8.8	0.4	603	20.4	120.43	0.04	0.001
Rusałka	37.0	1.9	9.0	8.72	0.9	774	17.5	3.61	0.04	0.04
Lusowskie	121.9	8.6	19.5	8.62	3	720	18.7	11.2	0.06	0.04
Wolsztyńskie	116.0	2.0	4.2	8.91	0.8	518	14.8	72.32	0.2	0.001
